# Physical activity as a protective factor for insomnia: systematic review, meta-analysis, and quality assessment of observational studies

**DOI:** 10.7189/jogh.16.04196

**Published:** 2026-08-28

**Authors:** Chao-Qun Xie, Fang-Fang Xie, Jia-He Cui, Ling-Jun Kong, Fei Yao, Min Fang

**Affiliations:** 1 Shuguang Hospital, Shanghai University of Traditional Chinese Medicine, Shanghai, China; 2 Shanghai Municipal Hospital of Traditional Chinese Medicine, Shanghai, China; 3 Shanghai University of Traditional Chinese Medicine, Shanghai, China

## Abstract

**Background:**

While meta-analyses of randomised controlled trials have demonstrated the therapeutic effects of physical activity (PA) on insomnia, the real-world associations between different levels and domains of PA and insomnia symptoms remain unclear. We aimed to examine these associations in the general population.

**Methods:**

We conducted a systematic review and meta-analysis of observational studies. We searched PubMed, MEDLINE, EMBASE, APA PsycINFO, CINAHL, and the Cochrane Library up to 5 August 2024, including studies that examined the association between PA and insomnia symptoms in the general population. We calculated pooled effect sizes using a random-effects model and performed subgroup analyses by PA intensity and domain. We assessed publication bias using funnel plots.

**Results:**

We included 27 risk estimates. Compared with low PA, high PA was not significantly associated with insomnia risk (odds ratio (OR) = 0.96; 95% confidence interval (CI) = 0.85–1.09). Moderate PA was associated with a 7% lower risk of insomnia (OR = 0.93; 95% CI = 0.87–1.00). Subgroup analyses showed significant variation across activity domains, with occupational PA associated with a 21% higher risk of insomnia (OR = 1.21; 95% CI = 1.02–1.43). There was no evidence of publication bias.

**Conclusions:**

Moderate levels of PA are associated with a modest reduction in insomnia risk, whereas occupational PA may increase the risk. These findings highlight the importance of considering both the intensity and domain of PA in relation to sleep health.

**Registration:**

PROSPERO: CRD42023417826.

Insomnia is a common mind-body disease that leads to health and functioning impairment [1]. Insomnia, characterised by its high prevalence and established role as a risk factor for numerous health conditions, imposes a substantial economic burden on healthcare and socioeconomic systems, with annual direct and indirect costs estimated at approximately USD 150 billion in the USA [2–4]. Pathophysiological studies of insomnia suggest that hyperarousal is a possible mechanism [5]. The first-line treatment, cognitive behavioural therapy for insomnia, suggests that the mechanism of action is consistent with models of sleep-wake regulation [6]. Cognitive behavioural therapy for insomnia is as effective as pharmacotherapy for acute treatment and more effective for long-term treatment [7]. However, its high costs greatly diminish the availability in communities running on limited budgets [8,9]. Approximately 40% of patients with insomnia do not reach sustained remission with these treatments. This estimate is based on clinical studies of cognitive behavioural therapy for insomnia, with a meta-analysis reporting a long-term remission rate of 41% (95% confidence interval (CI) = 31–53) [10]. The lack of accessible, rhythm-regulating and individualised treatment for insomnia has long been recognised [11–14].

Guidelines recommend physical activity (PA) as a treatment for insomnia, with the European guideline listing movement therapy, yoga, tai chi, and qigong as potential measures [15]. The World Health Organization advises adults aged 18–64 years to engage in at least 150 minutes of moderate-intensity or 75 minutes of high-intensity aerobic PA weekly, along with muscle-strengthening activities two days per week. Meta-analyses of randomised controlled trials (RCTs) have confirmed the therapeutic effect of PA on insomnia and provided specific recommendations [16,17].

However, RCTs may have several limitations. For example, some studies are conducted with relatively small sample sizes, which may limit statistical power and reduce the generalisability of findings. In addition, intervention designs are often highly structured and standardised, which may not fully reflect real-world conditions. Participant compliance can also vary, potentially affecting the effectiveness of the intervention. Furthermore, RCTs frequently involve highly selected populations, as strict inclusion and exclusion criteria (*e.g.* excluding individuals with comorbid conditions) are applied, which may limit the applicability of findings to broader populations.

In contrast, observational studies provide a broader perspective on the association between PA and insomnia in natural settings, reflecting real-world patterns of behaviour. They capture a wider range of PA domains, including leisure-time, occupational, and household activities, and therefore offer more generalisable evidence. Importantly, the relationship between PA and insomnia may not be linear, as higher levels of PA do not necessarily confer additional benefits and may, in some contexts, be associated with poorer sleep outcomes [18].

Conceptually, this relationship is likely to be complex and influenced by multiple pathways. Sleep might be improved with PA through physiological and psychological mechanisms, such as stress reduction, energy expenditure, and circadian rhythm regulation. At the same time, this association may be affected by confounding factors, including mental health status, lifestyle behaviours, and socioeconomic conditions. In addition, a bidirectional relationship is possible, whereby insomnia symptoms may reduce an individual’s likelihood of engaging in PA. Therefore, a synthesis of observational evidence is needed to better understand these real-world and potentially bidirectional associations and to complement findings from RCT-based meta-analyses.

We aimed to systematically review and meta-analyse observational studies to comprehensively assess the association between PA and insomnia in the general population, with a focus on real-world evidence and the gradient relationship.

## METHODS

In this systematic review and meta-analysis, we followed the PRISMA and adhered to the Meta-analysis of Observational Studies in Epidemiology guidelines [19]. We registered the predetermined methods online with PROSPERO (CRD42023417826).

### Data sources and searches

A research librarian searched PubMed, APA PsycINFO, MEDLINE, EMBASE, Cochrane Library, and CINAHL databases for English-language studies from inception to 5 August 2024 (eMethods in the **Online Supplementary Document**). We selected the search period 'from inception' to ensure a comprehensive retrieval of all relevant evidence. We applied the restriction to English-language studies based on quality considerations, as English-language journals in this field typically adhere to rigorous peer-review processes and standardised reporting guidelines, ensuring a higher baseline of methodological reliability for the included studies. Investigators manually reviewed reference lists of key studies and systematic reviews.

### Inclusion and exclusion criteria

Two authors (CQX and FFX) comprehensively and independently reviewed the articles. We included studies whose participants were adults (aged ≥18 years) with insomnia, as defined by established diagnostic criteria (*e.g.* DSM-5), standardised instruments (*e.g.* Pittsburgh sleep quality index, sleep diaries) or medical diagnosis, but with no other restrictions; studies that investigated the relationship between PA and insomnia; used observational studies in cohort or case-control designs; published odds ratios (ORs), relative risks (RRs), or hazard ratios (HRs); and reported a RR with its corresponding 95% CI or sufficient information to calculate these indices. We excluded studies reporting only absolute effect measures, such as risk differences, because it is strongly dependent on baseline risk and may not be comparable across heterogeneous populations; recruiting patients with secondary insomnia (*i.e.* insomnia due to psychiatric or physical comorbidity, or due to a medication or a substance such as alcohol); combined PA with other interventions such as diet, behavioural therapy, or antidepressant medication; and those that were classified as an editorial, RCT, review, meta-analysis, comment, news, letter, or practice guideline. We also excluded conference abstracts, editorials, commentaries, and qualitative studies because they typically do not provide sufficient methodological details or extractable quantitative data required for meta-analysis, and conference abstracts often represent preliminary findings that have not undergone full peer review. Investigators managed references in EndNote X9 software (Clarivate Analytics, Philadelphia, Pennsylvania, USA).

### Data extraction

Two authors (CQX and FFX) independently screened the titles and abstracts of all retrieved records and subsequently assessed the full texts of potentially eligible studies. They also independently extracted the data, using a structured form developed based on the study objectives and key variables of interest. Extracted information included study characteristics, participant details, PA measures, outcome definitions, confounders, and effect estimates (Table S1 in the **Online Supplementary Document**). Any disagreements were resolved through discussion and, when necessary, consultation with a third reviewer (FY). Two investigators (JHC and LJK) verified data for accuracy. We extracted all data directly from publications, and contacting authors for additional information was not necessary. During the screening process, we excluded studies that did not report sufficient data to derive effect estimates. When multiple estimates were available, we selected the most fully adjusted estimate for analysis.

In addition, based on the primary objective of this study – to investigate the association between PA and the risk of insomnia – we defined a single primary outcome, namely the risk of insomnia. We included studies that reported insomnia as a clinical diagnosis, assessed insomnia symptoms using validated instruments (*e.g.* the Pittsburgh Sleep Quality Index), or measured self-reported insomnia-related complaints. We excluded studies that did not provide a clear definition of insomnia or did not report effect estimates related to insomnia risk. Given the variability in outcome definitions across studies, we harmonised all measures as indicators of insomnia risk for pooled analysis. When multiple definitions were reported within a study, we prioritised the most clinically relevant or fully adjusted estimate.

### Risk of bias (quality) assessment

We rated the risk of bias (quality) of cohort and cross-sectional studies (based on comparability, outcome, and selection) as low (≤4) or high (5–9) using the Newcastle-Ottawa Scale [20].

### Strength of evidence

We assessed the strength of evidence using modified GRADE criteria [21]. Ratings were based on study limitations (low, medium, or high level), consistency (consistent, inconsistent, or unknown/not applicable), directness (direct or indirect), precision (precise or imprecise), and reporting bias (suspected or undetected). We assigned an overall grade of high, moderate, low, or insufficient by evaluating and weighing the combined results of the above domains. Two reviewers (CQX and FFX) assessed each domain for each outcome and determined an overall grade, with differences resolved by consensus.

### Data synthesis and analysis

We quantified the association between PA and insomnia symptoms using RR estimates (RRi), considering ORs, HRs, and RRs as comparable measures of association and intending to pool them together. This approach assumes that these measures approximate each other when the outcome is relatively uncommon, although differences in their mathematical properties should be considered when interpreting the results. The pooling of different effect measures (*i.e.* ORs, HRs, and RRs) may introduce some approximation error due to their differing statistical properties [22]. We preferentially extracted adjusted effect estimates from the original studies when available, as they account for potential confounding factors. If adjusted estimates were not reported, we used unadjusted estimates. We pooled together all selected effect estimates and treated them as equivalent measures of association. Otherwise, we calculated RRs and 95% CIs. We calculated the log-transformed RRi and its standard error (SE) as SE = (log (upper 95% CI limit of the RR) – log (RR)) / 1.96.

We used a random-effects model to calculate the pooled effect estimates, as we expected heterogeneity across studies due to differences in study populations, PA definitions, and outcome measurements [23]. This model accounts for both within- and between-study variability and is therefore appropriate for synthesising observational data. Weights for log (RRi) were calculated as wi = 1 / (si^2^ + t^2^), where si is the SE of log (RRi), and t^2^ is the restricted maximum likelihood estimate of the overall variance.

We assessed heterogeneity among studies using Q - and *I*^2^ - statistics. Although it is often considered that *I*^2^ values of 25% represent low, 50% moderate, and 75% high heterogeneity, these thresholds are approximate and should be interpreted with caution. Additionally, we assessed publication bias using funnel plots to detect asymmetry. We used Begg’s rank correlation and Egger’s linear regression tests as formal statistical methods to evaluate small-study effects [24,25]. We applied these methods when a sufficient number of studies were available (≥10 studies), as their reliability is limited when the number of studies is small. We set the statistical significance at *P* < 0.05.

In a sub-analysis, we explored the relationship between PA and insomnia symptoms across different subgroups, including sex (men, women), PA intensity (high, moderate, low) and PA categories (recreational, occupational, total). We selected these subgroup variables *a priori* based on their relevance in previous literature and their potential influence on both PA patterns and sleep outcomes. We conducted stratified analyses to explore potential sources of heterogeneity and to enhance the interpretability of the findings.

We used *R*, version 4.4.2 (R Core Team, Vienna, Austria) and Stata/Special Edition, version 16.1 (StataCorp LLC, College Station, Texas, USA) for all analyses. Given the potential for type I error due to multiple comparisons in the observational studies, the meta-analysis findings should be interpreted as exploratory.

## RESULTS

### Study characteristics

We conducted a comprehensive literature search, yielding a total of 10 811 studies from PubMed (n = 2,400), Medline (n = 1,191), EMBASE (n = 4,374), APA PsycINFO (n = 640), CINAHL (n = 684), and the Cochrane Library (n = 1,522). After the removal of duplicates and irrelevant studies, we included 19 eligible studies in the final analysis (Figure S1 in the **Online Supplementary Document**). All included studies were cohort studies [26–44], as no eligible case-control studies met the inclusion criteria, involving a total of 638 792 subjects and 90 507 insomnia cases. All included studies reported ORs as the measure of association.

### Study quality

We assessed the quality of the included studies using the Newcastle-Ottawa Scale. The average quality score was 5.3, indicating that the studies were of medium quality. Most of the studies were adjusted for potential confounders, such as smoking, alcohol consumption, and marital status (Table S2 in the **Online Supplementary Document**).

### Main efficacy meta-analysis

#### Overall analysis

The overall analysis of 27 risk estimates from the included studies did not reveal a statistically significant association between high levels of physical PA and reduced risk of insomnia compared to low levels of PA (Figure S2 in the **Online Supplementary Document**). The pooled OR was 0.96 (95% CI = 0.85–1.09), with substantial heterogeneity among studies (*I*^2^ = 74.0%; *P* = 0.000). This suggests that while there is a trend towards a reduced risk of insomnia with higher levels of PA, this association is not statistically significant and may be influenced by the substantial heterogeneity observed across studies.

#### Subgroup analysis by gender

We conducted a subgroup analysis stratified by gender to explore the differential effects of PA on insomnia risk (Figure S5 in the **Online Supplementary Document**). The analysis of seven risk estimates showed that the association between high PA and insomnia risk did not significantly differ between males (OR = 0.94; 95% CI = 0.80–1.11; *I*^2^ = 52.3%; *P* = 0.123) and females (OR = 1.03; 95% CI = 0.93–1.15; *I*^2^ = 52.2%; *P* = 0.099). The overall pooled OR for both genders was 1.01 (95% CI = 0.96–1.07; *I*^2^ = 45.5%; *P* = 0.088).

#### Subgroup analysis by PA domain

We further stratified the analysis by PA domain to assess the consistency of the results across different types of PA (Figure S8 in the **Online Supplementary Document**). The effects were the highest for occupational PA (OR = 1.21; 95% CI = 1.02–1.43; *I*^2^ = 0.0%; *P* = 0.704), followed by total PA (OR = 0.99; 95% CI = 0.90–1.09; *I*^2^ = 72.4%; *P* = 0.000) and recreational PA (OR = 0.85; 95% CI = 0.71–1.02; *I*^2^ = 77.6%; *P* = 0.000).

#### Subgroup analysis by PA levels

We conducted the analysis of different PA levels to determine the impact of varying levels of PA on insomnia risk (Figures S3 and S4 in the **Online Supplementary Document**). Compared to moderate PA, high PA was not significantly associated with insomnia (OR = 1.03, 95% CI = 0.97–1.09; *I*^2^ = 17.2%; *P* = 0.252). The association was not significantly different for males (OR = 1.03; 95% CI = 0.97–1.09; *I*^2^ = 0.0%; *P* = 0.444) and females (OR = 1.07; 95% CI = 1.00–1.14; *I*^2^ = 7.2%, *P* = 0.357) (Figure S6 in the **Online Supplementary Document**). For PA domain, the highest effect was in the occupational PA (OR = 1.42; 95% CI = 0.50–4.01), followed by recreational PA (OR *=* 0.93; 95% CI = 0.81–1.06; *I*^2^ = 22.2%; *P* = 0.252) and total PA (OR *=* 1.05; 95% CI = 1.01–1.09; *I*^2^ = 0.0%; *P* = 0.432) (Figure S9 in the **Online Supplementary Document**).

For moderate PA compared to low PA, the pooled OR was 0.93 (95% CI = 0.87–1.00; *I*^2^ = 38.0%; *P* = 0.057) (Figure S4 in the **Online Supplementary Document**). The effect was higher for males (OR = 0.80; 95% CI = 0.56–1.15; *I*^2^ = 81.7%; *P* = 0.004) than for females (OR = 0.96; 95% CI = 0.93–0.99; *I*^2^ = 0.0%; *P* = 0.516) (Figure S7 in the **Online Supplementary Document**). For PA domain, the effect was the highest for occupational PA (OR = 1.32; 95% CI = 0.47–3.72) followed by recreational PA (OR = 0.89; 95% CI = 0.79–0.99; *I*^2^ = 6.7%; *P* = 0.379) and total PA (OR = 0.95; 95% CI = 0.89–1.01; *I*^2^ = 55.1%; *P* = 0.029) (Figure S10 in the **Online Supplementary Document**).

#### Sensitivity analysis and publication bias

We performed a leave-one-out sensitivity analysis to assess the robustness of the pooled OR. The original random-effects meta-analysis yielded a pooled OR of 0.96 (95% CI = 0.85–1.09). After sequentially removing each study, the recalculated pooled ORs ranged from 0.93 to 0.97, with all estimates remaining within the original CI (Figure S11 in the **Online Supplementary Document**). The overall association remained similar in all iterations, supporting the robustness of the primary conclusion. We assessed publication bias using funnel plots, Begg, and Egger’s regression test. The funnel plot appeared symmetrical, suggesting no strong evidence of publication bias (Figure S12 in the **Online Supplementary Document**). However, given the relatively small number of included studies (n = 19), the statistical power of Begg’s and Egger’s tests is limited, and these results should be interpreted with caution (Figures S13 and S14 in the **Online Supplementary Document**).

## DISCUSSION

### Association between PA and insomnia risk

After reviewing 19 eligible observational studies, encompassing over 600,000 participants, we found that moderate levels of PA may offer significant benefits in reducing the risk of insomnia. However, there was no significant association between high levels of PA and a reduced risk of insomnia. The observational study designs used are particularly useful for assessing the real-world effects of PA as they provide insights from diverse populations, in contrast to the controlled environments of RCTs. Furthermore, we found that different types of PA had varying effects: recreational PA was associated with a decreased risk of insomnia, while occupational PA seemed linked to a higher risk. These findings suggest that the intensity and context of PA play a critical role in its effects on insomnia.

### Subgroup analysis of insomnia

Existing literature suggests that moderate PA may reduce insomnia risk, particularly in subgroups where high-intensity activity might not be ideal. Previous meta-analyses focusing on RCTs have consistently shown positive effects of aerobic exercise on insomnia [45–47], while observational studies, which reflect real-world conditions, exhibit more variability. This variability can be attributed to differences in methodology and the contexts in which PA occurs. Our study aligns with this literature; however, the observed association was modest and close to null, suggesting that it may be sensitive to residual heterogeneity and unmeasured confounding. Several factors may explain the observed discrepancies in the association between PA and insomnia. We found substantial heterogeneity across several analyses (*I*^2 ^> 70% in some comparisons), which may reflect differences in study populations, measurement methods, and adjustment strategies. For example, the included studies did not consistently control for potential confounders such as mental health history, lifestyle habits, and sleep disorders. Moreover, both PA and insomnia were assessed using different approaches across studies, including self-reported questionnaires and objective assessments [16,48], which may have further contributed to the variability in findings. Differences in age, sex, and socioeconomic background across study populations may also have influenced these associations. Although meta-regression could potentially help identify sources of heterogeneity, the relatively limited number of studies within subgroups reduced the statistical power and stability of such analyses. Therefore, we conducted stratified analyses by sex, PA intensity, and activity domain to partially explore these sources of variability. Taken together, these sources of variability may introduce uncertainty into the pooled estimates and partly explain the inconsistency in findings across studies. The study designs, which included cohort studies, allowed us to assess PA's real-world effects across diverse settings, though they also introduced the challenge of unmeasured confounders. Future studies should consider adjusting for a broader range of confounders, such as mental health conditions or lifestyle factors (*e.g.* smoking or alcohol consumption), which can influence both PA levels and insomnia symptoms.

### Biological mechanisms

Moderate-intensity PA, such as walking or light aerobics, may improve insomnia through several mechanisms. One explanation is that moderate PA can help regulate circadian rhythms, which is consistent with findings from several studies in our analysis [49]. These rhythms are crucial for maintaining healthy sleep patterns, and moderate PA has been shown to enhance circadian synchronisation, potentially facilitating sleep onset [50,51]. Additionally, moderate PA helps reduce stress and anxiety, both of which are key contributors to insomnia [52]. In general, PA triggers the release of endorphins, which improve mood and reduce mental stress, providing a direct pathway for improved sleep quality [53].

Our study suggests that PA’s effects are not uniform, and the context matters. Specifically, recreational PA, which is generally less stressful and more enjoyable, was associated with a reduced risk of insomnia [54]. In contrast, occupational PA, which often involves higher physical demands and work-related stress, appeared to be linked to a higher risk of insomnia [55]. However, this finding was based on a limited number of studies (n = 3) and should be considered hypothesis-generating rather than conclusive. These findings highlight the importance of PA context in understanding its impact on sleep.

One potential public health recommendation is to encourage more recreational PA in daily routines [56]. Specifically, clinicians can promote recreational PA as part of routine health counselling, particularly for individuals at risk of sleep disorders. Public health authorities could implement community-based programmes and awareness campaigns to promote enjoyable, moderate-intensity activities (*e.g.* walking or recreational sports) that help reduce stress and improve sleep without the negative effects associated with high-intensity or work-related PA. In addition, policymakers can support these efforts by improving access to safe and accessible spaces for PA, such as parks and recreational facilities. Furthermore, workplace wellness programmes could incorporate stress management strategies alongside occupational physical activities to minimise any negative impact on sleep quality. However, these implications should be interpreted considering several important limitations. Reverse causation cannot be excluded, as individuals with insomnia symptoms may be less likely to engage in regular PA due to fatigue, reduced motivation, or impaired daytime functioning. In addition, moderate PA may cluster with other healthy lifestyle behaviours, such as better diet, lower stress levels, and more regular daily routines. These co-occurring factors may contribute to improved sleep outcomes, making it difficult to isolate the independent effect of PA.

### Strengths and limitations

One of the major strengths of this meta-analysis is that we included a large and diverse sample of >600 000 participants, covering various age groups, genders, and socioeconomic backgrounds. This enhances the generalisability of the findings and increases their relevance to real-world populations. Second, the use of a random-effects model allowed us to account for variability across studies. We chose this model because it assumes that the true effect of PA on insomnia may vary across studies, and it weights the results accordingly. This approach ensures that our conclusions are robust, even in the face of methodological differences or heterogeneity between the included studies. However, this study has several limitations. First, the observational nature of the included studies prevents definitive causal inferences and reverse causation cannot be ruled out. While we found associations between PA and insomnia risk, these findings do not establish a causal relationship. Residual confounding is likely, as important factors such as mental health status, stress, socioeconomic status, chronic disease, and shift work were not consistently adjusted for across studies. Second, there is methodological variability across studies, including differences in how PA and insomnia were measured. Most studies assessed PA using self-reported measures with varying classification criteria and cut-points, while insomnia was defined using a variety of approaches, including symptom-based assessments, validated questionnaires, and clinical diagnoses. These inconsistencies may have contributed to heterogeneity in the results and could affect the comparability of findings across studies. Third, potential confounding factors, such as mental health conditions, lifestyle habits (*e.g.* smoking and alcohol use), and other sleep-related disorders, were not consistently controlled for, raising the possibility that the observed associations may have been influenced by unmeasured variables. Consistent with the risk of bias assessment, although most included studies were of moderate to high quality, residual confounding and limitations in measurement may still have influenced the observed associations. In addition, although the analytical framework allowed for pooling different effect measures (ORs, HRs, and RRs), all included studies reported ORs. Therefore, no transformation between different effect measures was required. However, ORs may be less intuitive to interpret than RRs, particularly when the outcome is not rare. Finally, although the presentation of insomnia may vary across demographic groups, we focused on the general adult population to capture overall associations in real-world settings, and the findings should be interpreted with consideration of potential population differences.

### Implications

Future research should further examine the relationship between PA and insomnia in more diverse populations, particularly in underrepresented settings such as low- and middle-income countries. Greater use of standardised and objective measures would improve comparability across studies, while longitudinal and interventional designs may help clarify the directionality and underlying mechanisms of this association.

## CONCLUSIONS

Moderate-intensity PA was significantly associated with a lower risk of insomnia compared to low-intensity PA. These findings suggest that moderate PA may be associated with a reduced risk of insomnia in the general population. However, given the observational nature of the evidence, causal inferences cannot be established. Our findings add to the growing body of evidence on non-pharmacological approaches to insomnia and highlight the potential role of moderate PA in sleep health. Future research, particularly well-designed prospective and interventional studies, is needed to further clarify these associations and their potential implications for public health.

## Additional material


Online Supplementary Document
