# Supplementary material for: Physical activity as a protective factor for insomnia: systematic review, meta-analysis, and quality assessment of observational studies
Source: J Glob Health. 2026 Aug 28;16:04196. doi: 10.7189/jogh.16.04196 (PMC13521491; doi:10.7189/jogh.16.04196)

Supplementary appendix
Supplement to:
Physical activity as a protective factor for insomnia: systematic review, meta-analysis and quality assessment of observational studies

eMethods: Search Strategy
Table S1. Characteristics of the 19 studies on physical activity and insomnia risk included in the meta-analysis
Table S2. Strength of Evidence
Figure S1. PRISMA flow diagram of identification and selection of eligible studies
Figure S2. Forest plot of a random effects meta-analysis including 27 risk estimates of insomnia for a high versus low level of PA
Figure S3. Forest plot of a random effects meta-analysis including 17 risk estimates of insomnia for a high versus moderate level of PA
Figure S4. Forest plot of a random effects meta-analysis including 17 risk estimates of insomnia for a moderate versus low level of PA
Figure S5. Forest plot of a random effects meta-analysis including 7 risk estimates of insomnia for a high versus low level of PA, grouped by gender
Figure S6. Forest plot of a random effects meta-analysis including 7 risk estimates of insomnia for a high vs moderate level of PA, grouped by gender
Figure S7. Forest plot of a random effects meta-analysis including 7 risk estimates of insomnia for a moderate vs low level of PA, grouped by gender
Figure S8. Forest plot of a random effects meta-analysis including 27 risk estimates of insomnia for a high versus low level of PA, grouped by PA domain
Figure S9. Forest plot of a random effects meta-analysis including 17 risk estimates of insomnia for a high vs moderate level of PA, grouped by PA domain
Figure S10. Forest plot of a random effects meta-analysis including 17 risk estimates of insomnia for a moderate vs low level of PA, grouped by PA domain
Figure S11. Leave-One-Out Sensitivity Analysis
Figure S12. Standardized Funnel plot corresponding to the main random-effects meta-analysis
Figure S13. Standardised Begg's regression test corresponding to the main random-effects meta-analysis
Figure S14. Standardised Egger's regression test corresponding to the main random-effects meta-analysis

eMethods: Search Strategy

PubMed：

exercise：

(((((((((((((((((((((((((exercise) OR (Exercises, Physical)) OR (Physical Activities)) OR (Exercise, Physical)) OR (Activities, Physical)) OR (Physical Exercises)) OR (Activity, Physical)) OR (Exercises)) OR (Physical Activity)) OR (Physical Exercise)) OR (Acute Exercise)) OR (Acute Exercises)) OR (Exercise, Acute)) OR (Exercises, Acute)) OR (Training, Exercise)) OR (Exercise Trainings)) OR (Exercise Training)) OR (Trainings, Exercise)) OR (Exercises, Isometric)) OR (Isometric Exercises)) OR (Exercise, Isometric)) OR (Isometric Exercise)) OR (Aerobic Exercise)) OR (Exercises, Aerobic)) OR (Exercise, Aerobic)) OR (Aerobic Exercises)

insomnia：

((((((((((((((((((((((((((insomnia) OR (Nonorganic Insomnia)) OR (Nonorganic)) OR (Insomnia, Transient)) OR (Transient Insomnia)) OR (Chronic Insomnia)) OR (Insomnia, Chronic)) OR (DIMS (Disorders of Initiating and Maintaining Sleep))) OR (Disorders of Initiating and Maintaining Sleep)) OR (Awakening, Early)) OR (Early Awakening)) OR (Insomnia, Rebound)) OR (Rebound Insomnia)) OR (Insomnias)) OR (Insomnia Disorders)) OR (Sleeplessness)) OR (Insomnia Disorder)) OR (Sleep Initiation Dysfunctions)) OR (Dysfunctions, Sleep Initiation)) OR (Dysfunction, Sleep Initiation)) OR (Sleep Initiation Dysfunction)) OR (Insomnia, Psychophysiological)) OR (Psychophysiological Insomnia)) OR (Primary Insomnia)) OR (Insomnia, Primary)) OR (Secondary Insomnia)) OR (Insomnia, Secondary)

exercise and insomnia:

((((((((((((((((((((((((((exercise) OR (Exercises, Physical)) OR (Physical Activities)) OR (Exercise, Physical)) OR (Activities, Physical)) OR (Physical Exercises)) OR (Activity, Physical)) OR (Exercises)) OR (Physical Activity)) OR (Physical Exercise)) OR (Acute Exercise)) OR (Acute Exercises)) OR (Exercise, Acute)) OR (Exercises, Acute)) OR (Training, Exercise)) OR (Exercise Trainings)) OR (Exercise Training)) OR (Trainings, Exercise)) OR (Exercises, Isometric)) OR (Isometric Exercises)) OR (Exercise, Isometric)) OR (Isometric Exercise)) OR (Aerobic Exercise)) OR (Exercises, Aerobic)) OR (Exercise, Aerobic)) OR (Aerobic Exercises)) AND (((((((((((((((((((((((((((insomnia) OR (Nonorganic Insomnia)) OR (Nonorganic)) OR (Insomnia, Transient)) OR (Transient Insomnia)) OR (Chronic Insomnia)) OR (Insomnia, Chronic)) OR (DIMS (Disorders of Initiating and Maintaining Sleep))) OR (Disorders of Initiating and Maintaining Sleep)) OR (Awakening, Early)) OR (Early Awakening)) OR (Insomnia, Rebound)) OR (Rebound Insomnia)) OR (Insomnias)) OR (Insomnia Disorders)) OR (Sleeplessness)) OR (Insomnia Disorder)) OR (Sleep Initiation Dysfunctions)) OR (Dysfunctions, Sleep Initiation)) OR (Dysfunction, Sleep Initiation)) OR (Sleep Initiation Dysfunction)) OR (Insomnia, Psychophysiological)) OR (Psychophysiological Insomnia)) OR (Primary Insomnia)) OR (Insomnia, Primary)) OR (Secondary Insomnia)) OR (Insomnia, Secondary))

Medline
1: MH=(insomnia)
2: ((((((((((((((((((((((((TS=(Nonorganic Insomnia)) OR TS=(Nonorganic)) OR TS=(Insomnia, Transient)) OR TS=(Transient Insomnia)) OR TS=(Chronic Insomnia)) OR TS=(Insomnia, Chronic)) OR TS=(DIMS (Disorders of Initiating and Maintaining Sleep))) OR TS=(Disorders of Initiating and Maintaining Sleep)) OR TS=(Awakening, Early; Early Awakening)) OR TS=(Insomnia, Rebound)) OR TS=(Rebound Insomnia)) OR TS=(Insomnias)) OR TS=(Insomnia Disorders)) OR TS=(Sleeplessness)) OR TS=(Insomnia Disorder)) OR TS=(Sleep Initiation Dysfunctions)) OR TS=(Dysfunctions, Sleep Initiation)) OR TS=(Dysfunction, Sleep Initiation)) OR TS=(Sleep Initiation Dysfunction)) OR TS=(Insomnia, Psychophysiological)) OR TS=(Psychophysiological Insomnia)) OR TS=(Primary Insomnia)) OR TS=(Insomnia, Primary)) OR TS=(Secondary Insomnia)) OR TS=(Insomnia, Secondary)
3: #2 OR #1
4: MH=(exercise)
5: (((((((((((((((((((((((TS=(Exercises, Physical)) OR TS=(Physical Activities)) OR TS=(Exercise, Physical)) OR TS=(Activities, Physical)) OR TS=(Physical Exercises)) OR TS=(Activity, Physical)) OR TS=(Exercises; Physical Activity)) OR TS=(Physical Exercise)) OR TS=(Acute Exercise)) OR TS=(Acute Exercises)) OR TS=(Exercise, Acute)) OR TS=(Exercises, Acute)) OR TS=(Training, Exercise)) OR TS=(Exercise Trainings)) OR TS=(Exercise Training)) OR TS=(Trainings, Exercise)) OR TS=(Exercises, Isometric)) OR TS=(Isometric Exercises)) OR TS=(Exercise, Isometric)) OR TS=(Isometric Exercise)) OR TS=(Aerobic Exercise)) OR TS=(Exercises, Aerobic)) OR TS=(Exercise, Aerobic)) OR TS=(Aerobic Exercises)
6: #4 OR #5
7: #3 AND #6

Cochrane Library
#1 MeSH descriptor: [Sleep Initiation and Maintenance Disorders] explode all trees
#2 (insomnia):ti,ab,kw (Word variations have been searched)
#3 (Nonorganic Insomnia):ti,ab,kw (Word variations have been searched)
#4 (Nonorganic):ti,ab,kw (Word variations have been searched)
#5 (Insomnia, Transient):ti,ab,kw (Word variations have been searched)
#6 (Transient, Insomnia):ti,ab,kw (Word variations have been searched)
#7 (Chronic Insomnia):ti,ab,kw (Word variations have been searched)
#8 (Insomnia, Chronic):ti,ab,kw (Word variations have been searched)
#9 (DIMS (Disorders of Initiating and Maintaining Sleep)):ti,ab,kw (Word variations have been searched)
#10 (Disorders of Initiating and Maintaining Sleep):ti,ab,kw (Word variations have been searched)
#11 (Awakening, Early):ti,ab,kw (Word variations have been searched)
#12 (Early Awakening):ti,ab,kw (Word variations have been searched)
#13 (Insomnia, Rebound):ti,ab,kw (Word variations have been searched)

#14 (Rebound Insomnia):ti,ab,kw (Word variations have been searched)
#15 (Insomnias):ti,ab,kw (Word variations have been searched)
#16 (Insomnia Disorders):ti,ab,kw (Word variations have been searched)
#17 (Sleeplessness):ti,ab,kw (Word variations have been searched)
#18 (Insomnia Disorder):ti,ab,kw (Word variations have been searched)
#19 (Sleep Initiation Dysfunctions):ti,ab,kw (Word variations have been searched)
#20 (Dysfunctions, Sleep Initiation):ti,ab,kw (Word variations have been searched)
#21 (Sleep Initiation Dysfunction):ti,ab,kw (Word variations have been searched)
#22 (Insomnia, Psychophysiological):ti,ab,kw (Word variations have been searched)
#23 (Psychophysiological Insomnia):ti,ab,kw (Word variations have been searched)
#24 (Primary Insomnia):ti,ab,kw (Word variations have been searched)
#25 (Insomnia, Primary):ti,ab,kw (Word variations have been searched)
#26 (Secondary Insomnia):ti,ab,kw (Word variations have been searched)
#27 (Insomnia, Secondary):ti,ab,kw (Word variations have been searched)
#28 MeSH descriptor: [Exercise] explode all trees
#29 (exercise):ti,ab,kw (Word variations have been searched)
#30 (Exercises, Physical):ti,ab,kw (Word variations have been searched)
#31 (Physical Activities):ti,ab,kw (Word variations have been searched)
#32 (Exercise, Physical):ti,ab,kw (Word variations have been searched)
#33 (Activities, Physical):ti,ab,kw (Word variations have been searched)
#34 (Physical Exercises):ti,ab,kw (Word variations have been searched)
#35 (Activity, Physical):ti,ab,kw (Word variations have been searched)
#36 (Exercises):ti,ab,kw (Word variations have been searched)
#37 (Physical Activity):ti,ab,kw (Word variations have been searched)
#38 (Physical Exercise):ti,ab,kw (Word variations have been searched)
#39 (Acute Exercise):ti,ab,kw (Word variations have been searched)
#40 (Acute Exercises):ti,ab,kw (Word variations have been searched)
#41 (Exercise, Acute):ti,ab,kw (Word variations have been searched)
#42 (Exercises, Acute):ti,ab,kw (Word variations have been searched)
#43 (Training, Exercise):ti,ab,kw (Word variations have been searched)
#44 (Exercise Trainings):ti,ab,kw (Word variations have been searched)
#45 (Exercise Training):ti,ab,kw (Word variations have been searched)
#46 (Trainings, Exercise):ti,ab,kw (Word variations have been searched)
#47 (Exercises, Isometric):ti,ab,kw (Word variations have been searched)
#48 (Isometric Exercises):ti,ab,kw (Word variations have been searched)
#49 (Exercise, Isometric):ti,ab,kw (Word variations have been searched)
#50 (Isometric Exercise):ti,ab,kw (Word variations have been searched)
#51 (Aerobic Exercise):ti,ab,kw (Word variations have been searched)
#52 (Exercises, Aerobic):ti,ab,kw (Word variations have been searched)
#53 (Exercise, Aerobic):ti,ab,kw (Word variations have been searched)
#54 (Aerobic Exercises):ti,ab,kw (Word variations have been searched)
#55 #1 OR #2 OR #3 OR #4 OR #5 OR #6 OR #7 OR #8 OR #9 OR #10 OR #11 OR #12 OR #13 OR #14 OR #15 OR #16 OR #17 OR #18 OR #19 OR #20 OR #21 OR #22 OR #23 OR #24 OR #25 OR #26 OR #27

#56 #28 OR #29 OR #30 OR #31 OR #32 OR #33 OR #34 OR #35 OR #36 OR #37 OR #38 OR #39 OR #40 OR #41 OR #42 OR #43 OR #44 OR #45 OR #46 OR #47 OR #48 OR #49 OR #50 OR #51 OR #52 OR #53 OR #54
#57 #55 AND #56

Table S1. Characteristics of the 19 studies on physical activity and insomnia risk included in the meta-analysis

**Authors,year,gender**	**Study design**	**Region**	**Subjects**	**Cases**	**insomnia category**	**PA domain**	**Effect size (RR/OR/HR)**	**Low PA defined by**	**High PA defined by**	**Adjustment factors (excluding age and sex)**
Zheng, B.2017 [26]<br>Men<br>women	Cross-Sectional Study	China	452024	23444<br>48224	AIS	Total	1.01(0.97,1.06)<br>1.02(0.98,1.05)	no activity	High PA	Adjusted for age, geographic region, sociodemographic factors (education, marital status, income), body mass index, lifestyle factors (smoking, alcohol, tea consumption, and diet), mental health status, and menopausal status (women only).
Xu, H.2023[27]<br>Men and women	Cross-Sectional Study	China	8095	1859	AIS	Recreational	0.79(0.65,0.95)	low exercise	large amount of exercise	none
Werneck, A. O.2018 [28]	Cross-Sectional Study	Brazil	48559<br>52280	3438<br>8185	insomnia questions	Total	0.72(0.52,0.99)<br>1.06(0.88,1.27)	0 min per week	⩾420 min per week	Adjusted for age, race, geographic region, urbanicity,

Men<br>women										physical activity and sedentary behaviour, and dietary factors.
Toubasi, A. A.2023 [29]<br>Men and women	Cross-Sectional Study	Middle East	253<br>256	314	ISI	Total	1.47(0.73,2.96)	Inactive (not reach 600 MET minute/week,)	active(equal or higher than 600 MET minute/week.)	none
Sporndly-Nees, Soren 2017 [30]<br>Men and women	Cross-Sectional Study	Sweden	3800	173	ICSD-2	Recreational	0.3(0.16,0.54)	low PA	High PA	Adjusted for age, body mass index, physical activity, snoring, smoking, alcohol consumption, education, and psychological distress.
Skarpsno, E. S.2018<br>Men [31]<br>women	Cross-Sectional Study	Norway	9622<br>11347	374<br>632	DSM-5	Recreational	0.98(0.75,1.27)<br>1.09(0.94,1.26)	inactive(no light or no hard activity per week)	High PA(any light and ≥1 h hard activity per week)	Adjusted for age, body mass index, education, smoking, alcohol consumption, shift work, mental health status, and physical activity.

Skarpsno, Eivind Schjelderup.2019 [32] Men and women	Cross-Sectional Study	Denmark	678	98	insomnia questions	Recreational<br>Occupational	1.05(0.68,1.64)<br>1.22(0.76,1.94)	< 52 min/day<br>< 76 min/day	⩾ 52 min/day<br>⩾ 76 min/day	Adjusted for age, sex, body mass index, smoking, occupational factors (work sector and shift work), antidepressant use, pain, and physical activity.
Skarpsno, Eivind Schjelderup 2018 [33] Men and women	Cross-Sectional Study	Denmark	650	92	insomnia questions	Total<br>Recreational<br>Occupational	1.17(0.43,3.16)<br>1.03(0.41,2.59)<br>1.87(0.66,5.31)	very low PA 7-38min<br>very low PA 6-49 min	high PA 71-184 min<br>high PA 103-235 min	Adjusted for age, sex, body mass index, pain, alcohol consumption, smoking, occupational factors (work type and shift work), and antidepressant use.
Seol, J.2021 [34] Men and women	Cross-Sectional Study	Japan	988	337	PSQI, with a cut-off of 5/6	Total	0.69(0.48,1.00)	PASE score, 0–94.3 points	PASE score,140.6–462.3 points	Adjusted for age, sex, body mass index, hypertension medication use,

										hypnotic use, smoking, alcohol consumption, depressive symptoms, and social relationship factors.
Paparrigopoulos, T.2010 [35] Men and women	Cross-Sectional Study	Greece	483 522	94 160	AIS	Total	1.42(1.01,2.03)	IPAQ less than 30	IPAQ no less than 30	Adjusted for sociodemographic factors (age, sex, marital status, socioeconomic status, education), lifestyle factors (alcohol use, smoking, physical activity), and health-related factors (chronic disease, mental health, body mass index, and prior hospitalisations).
Liu, Z.2022 [36] Men and women	Cross-Sectional Study	China	179	41	ISI	Total	0.22(0.06,0.88)	less than 30 min per day	more than 60 min per day	none

Lim, M.2020 [37]<br>Men and women	Cross-Sectional Study	Korean	9788	890	ISI	Recreational<br>Occupational	1.37(1.13,1.67)<br>1.19(0.99,1.42)	low PA<br>low PA	High PA<br>High PA	Adjusted for age, sex, sociodemographic factors (education, marital status, income), lifestyle factors (smoking, alcohol, caffeine), occupational factors (job type, work schedule, notice time), mental health conditions (depression, anxiety, and post-traumatic stress disorder).
Kline, C. E. [38]<br>women	Cross-Sectional Study	USA	339	331	ISQ	Recreational	0.26(0.08,0.81)	Consistently Low	Consistently High	Adjusted for age, race/ethnicity, sociodemographic factors (marital status, education, employment), lifestyle factors (smoking,

										caffeine, alcohol), medication use, menopausal status, vasomotor symptoms, and body mass index.
Kitano, N.2014 [39] Men and women	Cross-Sectional Study	Japan	421	135	DIS	Recreational	0.67(0.18,2.45)	low PA	High PA	Adjusted for age, sex, hypnotic use, body mass index, and dairy consumption.
Inoue, S.2013 [40] Men and women	Cross-Sectional Study	Japan	10211	None	DIS	Recreational	0.74(0.58,0.94)	no activity	5 or more days/week	Adjusted for age, sex, body mass index, smoking, alcohol consumption, socioeconomic status, and mood status.
	Cohort study	Japan	3697	None	DIS	Recreational	0.72(0.49,1.06)	no activity	6 or more days/week	Adjusted for age, sex, body mass index, smoking, alcohol consumption, socioeconomic

										status, and mood status.
Chen, L. J.2017 [41] Men and women	Cross-Sectional Study	Taiwan, China	12728	845	ICD-9	Recreational	0.82(0.7,0.94)	inactive: 0-999 kcal/wk	active: 1000+ kcal/wk	Adjusted for sociodemographic and health-related variables, with additional adjustment for smoking status.
Endeshaw, Y. W.2016 [42] Men and women	Cross-Sectional Study	USA	3776434	832	DIS	Total	0.64(0.5,0.81)	no activity	None	Adjusted for age, sex, sociodemographic factors (race, marital status, education, income, neighborhood status), health status, and physical function.
Gubelmann, C.2018 [43] Men and women	Cross-Sectional Study	Switzerland	2649	None	ISI	Total	1.56(0.98,2.48)	low PA	High PA	Adjusted for age, sex, self-rated health, alcohol consumption,

										medication use, and occupational status.
Hartescu, I.2019 [44]<br>Men<br>women	Cross-Sectional Study	South Africa, Australia, China, South Korea and the UK	2227<br>7011	None	DSM-5	Total	1.31(1.02,1.69)	76-149 min per week	>300 min per week	Adjusted for age, sex, lifestyle factors (smoking, alcohol, caffeine), health status, medication use, socioeconomic variables, and country.

Abbreviations: AIS, Athens Insomnia Scale; ISI, Insomnia Severity Index; ICSD-2, International Classification of Sleep Disorders, 2nd edition; DSM-5, Diagnostic and Statistical Manual of Mental Disorders, 5th edition; PSQI, Pittsburgh Sleep Quality Index; DIS, Diagnostic Interview Schedule; ISQ, Insomnia Screening Questionnaire; PASE, Physical Activity Scale for the Elderly; IPAQ, International Physical Activity Questionnaire; MET, metabolic equivalent of task; PA, physical activity; RR, relative risk.

Table S2. Strength of Evidence

No.	First author, Year, country	Selection (Max, score4)	Comparability (Max, score2)	Outcome (max, score3)	Total (max, score9)	Quality of studies
1	Zheng, B.2017	3	1	1	5	Medium
2	Xu, H.2023	3	1	0	4	Medium
3	Werneck, A. O.2018	3	1	0	4	Medium
4	Toubasi, A. A.2023	3	1	1	5	Medium
5	Sporndly-Nees, Soren 2017	3	1	2	6	Medium
6	Skarpsno, E. S.2018	3	1	2	6	Medium
7	Skarpsno, Eivind Schjelderup.2019	3	1	0	4	Medium
8	Skarpsno, Eivind Schjelderup 2018	3	1	2	6	Medium
9	Seol, J.2021	3	1	2	6	Medium
10	Paparrigopoulos, T.2010	3	2	0	5	Medium
11	Liu, Z.2022	3	2	0	5	Medium
12	Lim, M.2020	3	2	0	5	Medium
13	Kline, C. E.2013	3	2	1	6	Medium
14	Kitano, N.2014	3	1	0	4	Medium
15	Inoue, S.2013	2	1	2	5	Medium
16	Chen, L. J.2017	4	1	3	8	High
17	Endeshaw, Y. W.2016	4	1	0	5	Medium
18	Gubelmann, C.2018	4	1	1	6	Medium
19	Hartescu, I.2019	4	1	0	5	Medium

Figure S1. PRISMA flow diagram of identification and selection of eligible studies

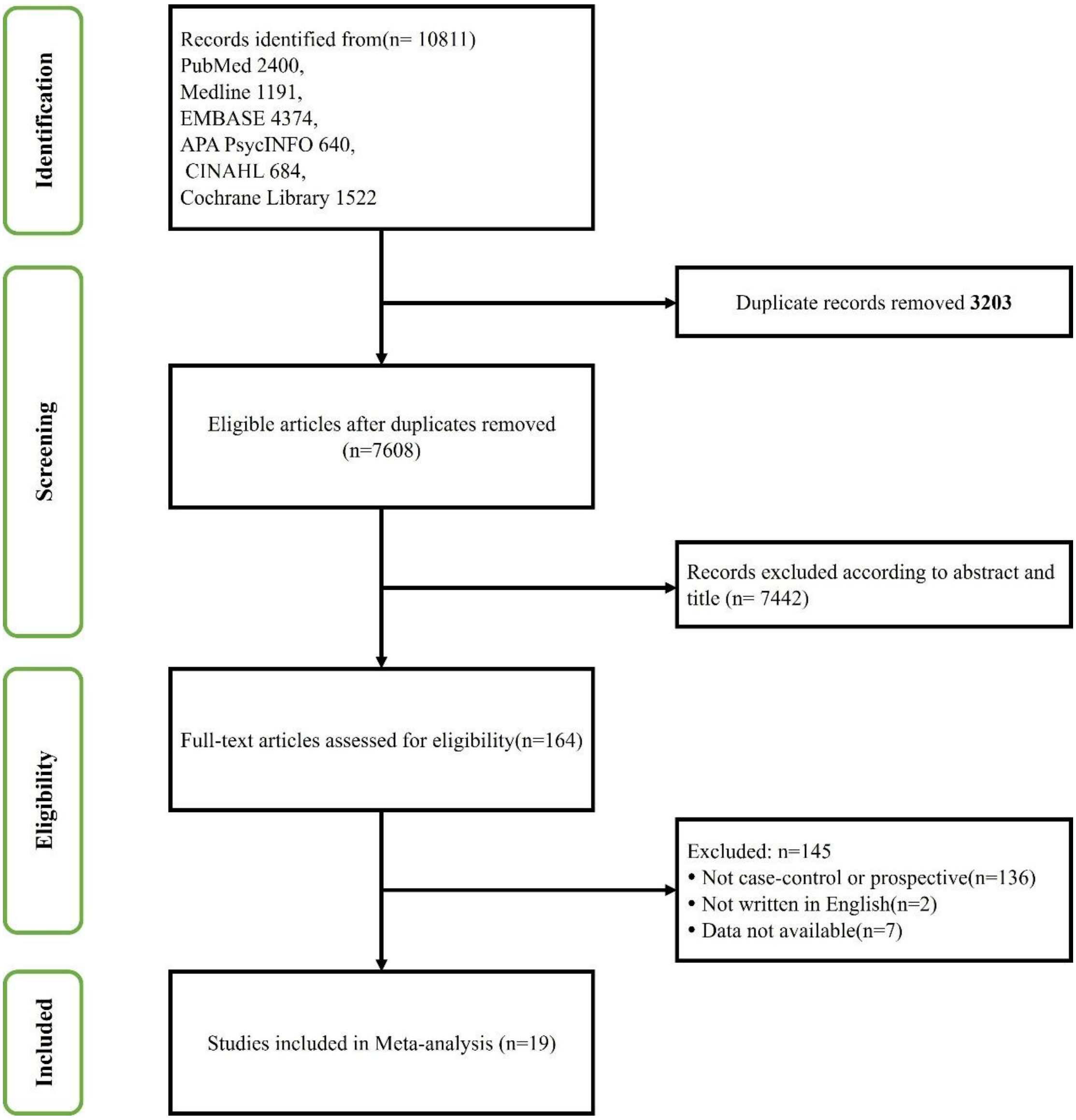

Figure S2. Forest plot of a random effects meta-analysis including 27 risk estimates of insomnia for a high versus low level of PA

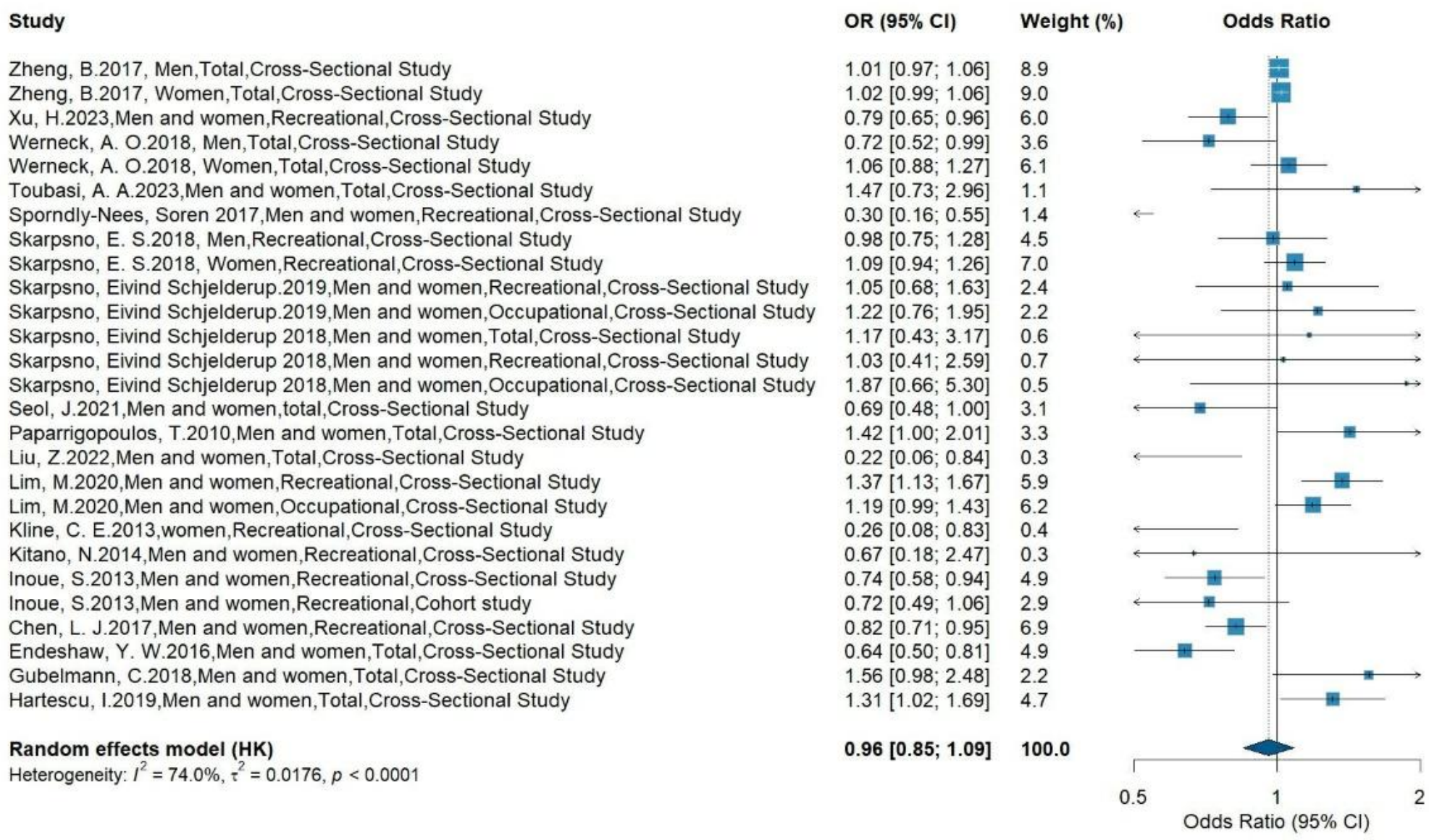

Abbreviations: OR, odds ratio; CI, confidence interval; PA, physical activity.

Figure S3. Forest plot of a random effects meta-analysis including 17 risk estimates of insomnia for a high versus moderate level of PA

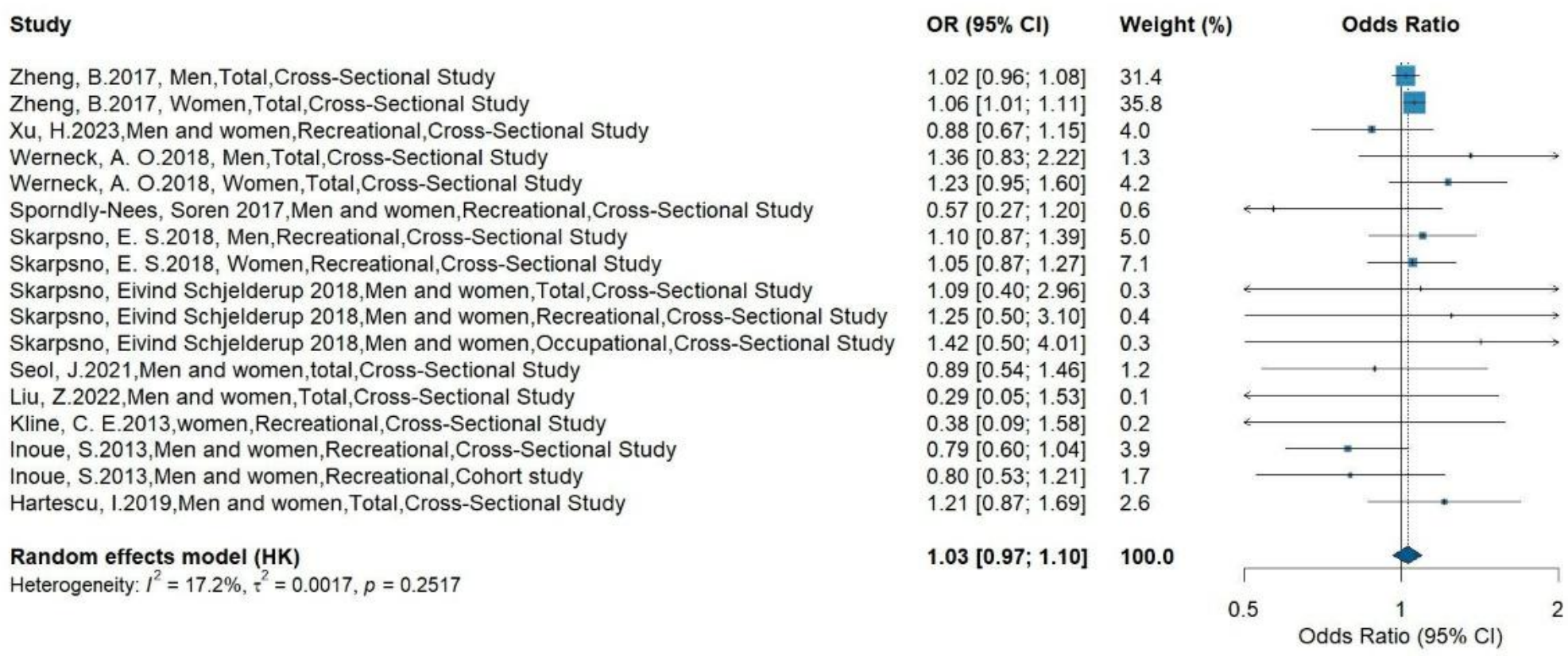

Abbreviations: OR, odds ratio; CI, confidence interval.

Figure S4. Forest plot of a random effects meta-analysis including 17 risk estimates of insomnia for a moderate versus low level of PA

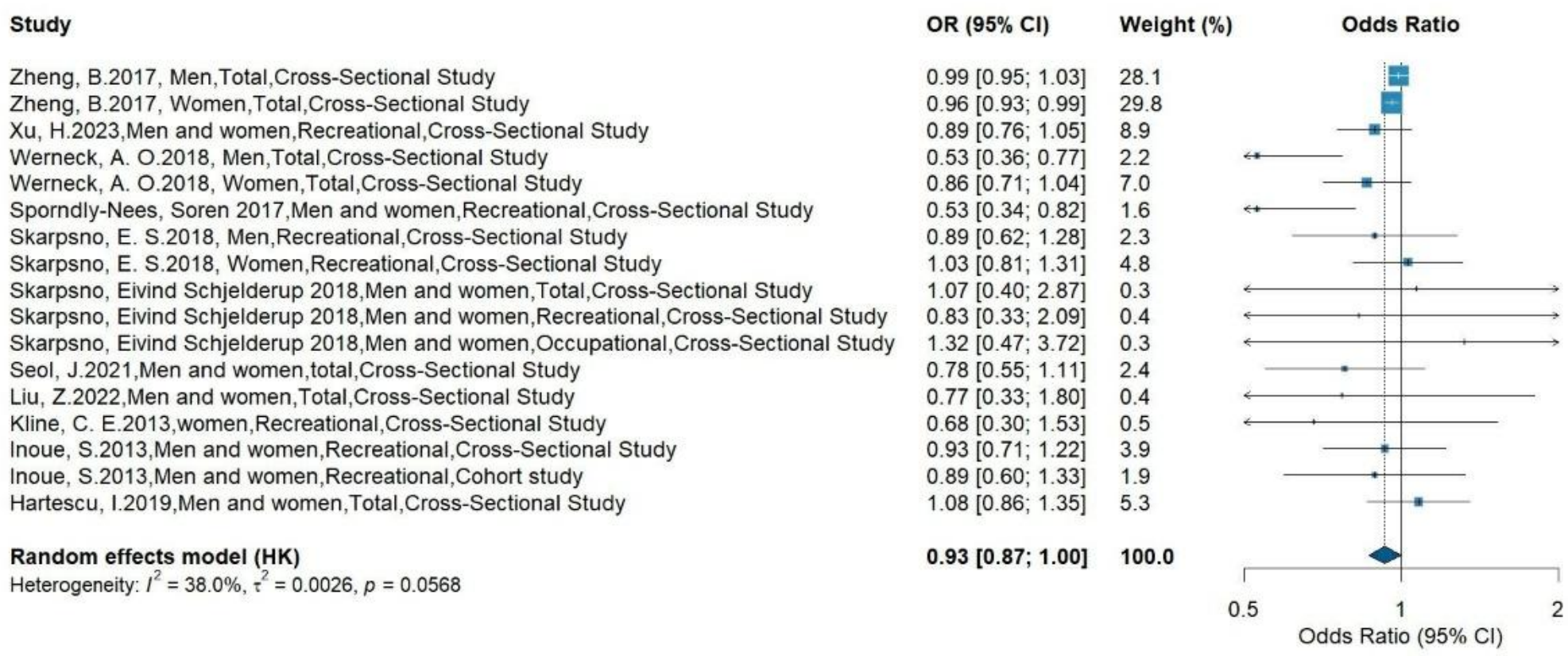

Abbreviations: OR, odds ratio; CI, confidence interval.

Figure S5. Forest plot of a random effects meta-analysis including 7 risk estimates of insomnia for a high versus low level of PA, grouped by gender

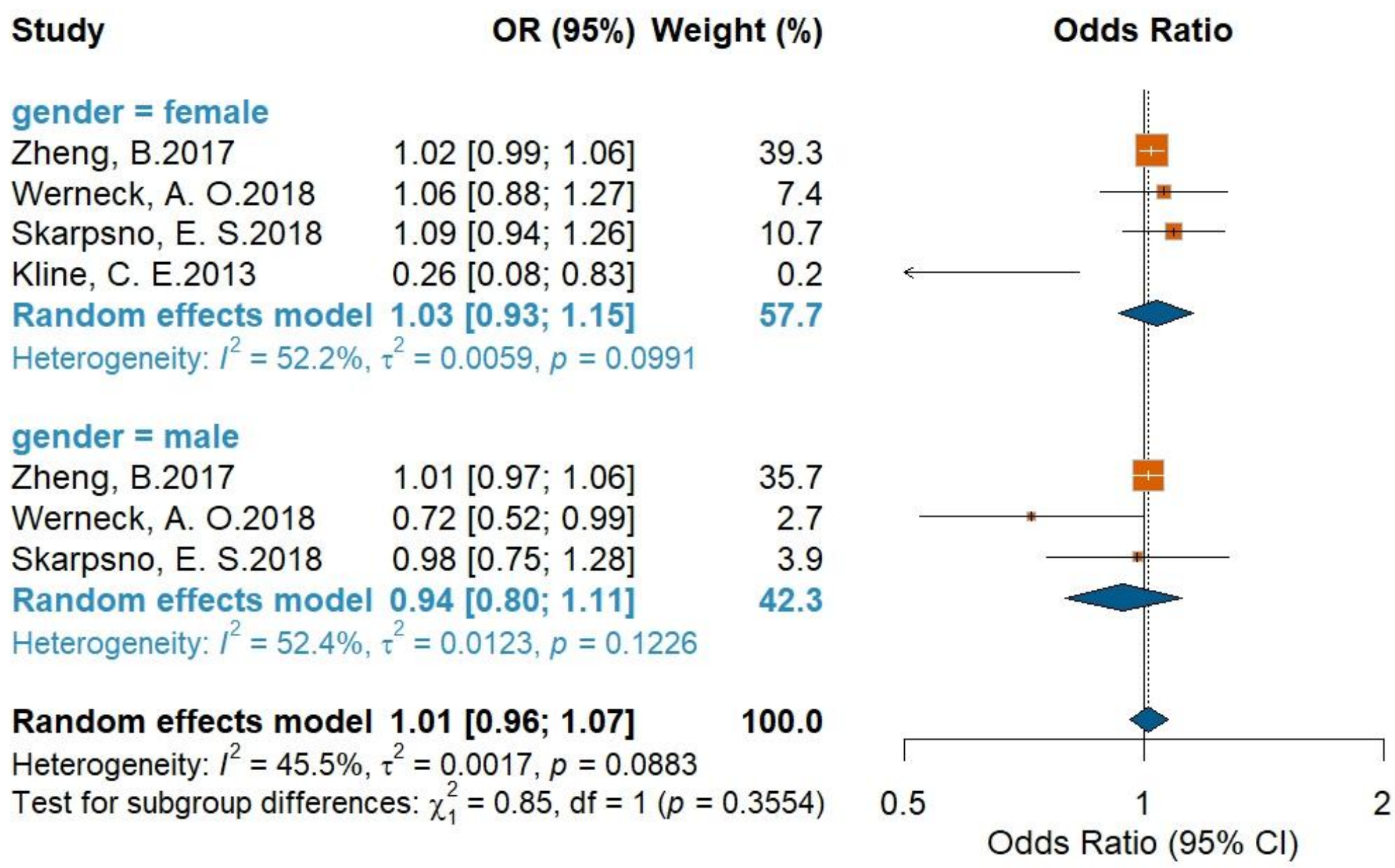

Abbreviations: OR, odds ratio; CI, confidence interval.

Figure S6. Forest plot of a random effects meta-analysis including 7 risk estimates of insomnia for a high vs moderate level of PA, grouped by gender

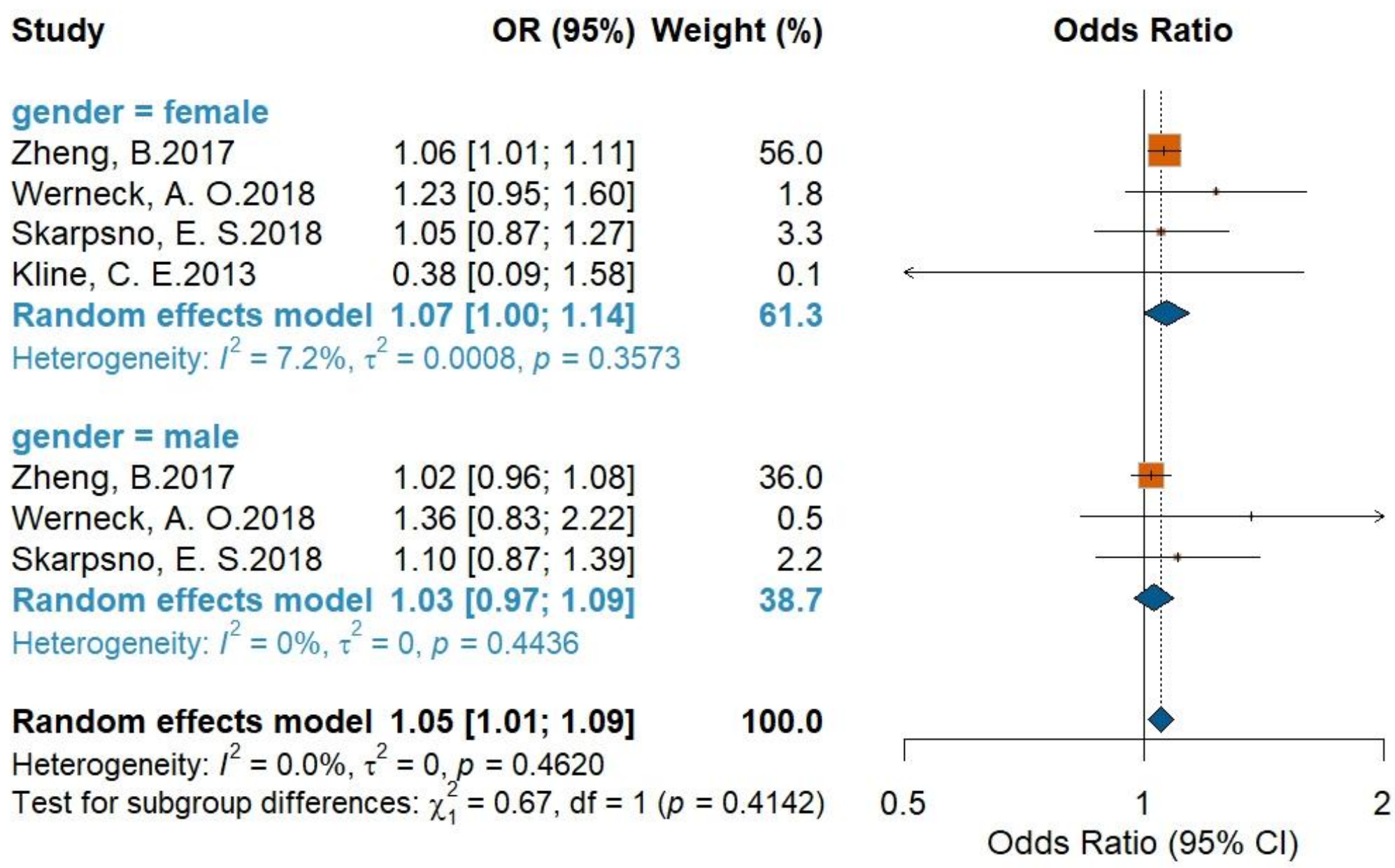

Abbreviations: OR, odds ratio; CI, confidence interval.

Figure S7. Forest plot of a random effects meta-analysis including 7 risk estimates of insomnia for a moderate vs low level of PA, grouped by gender

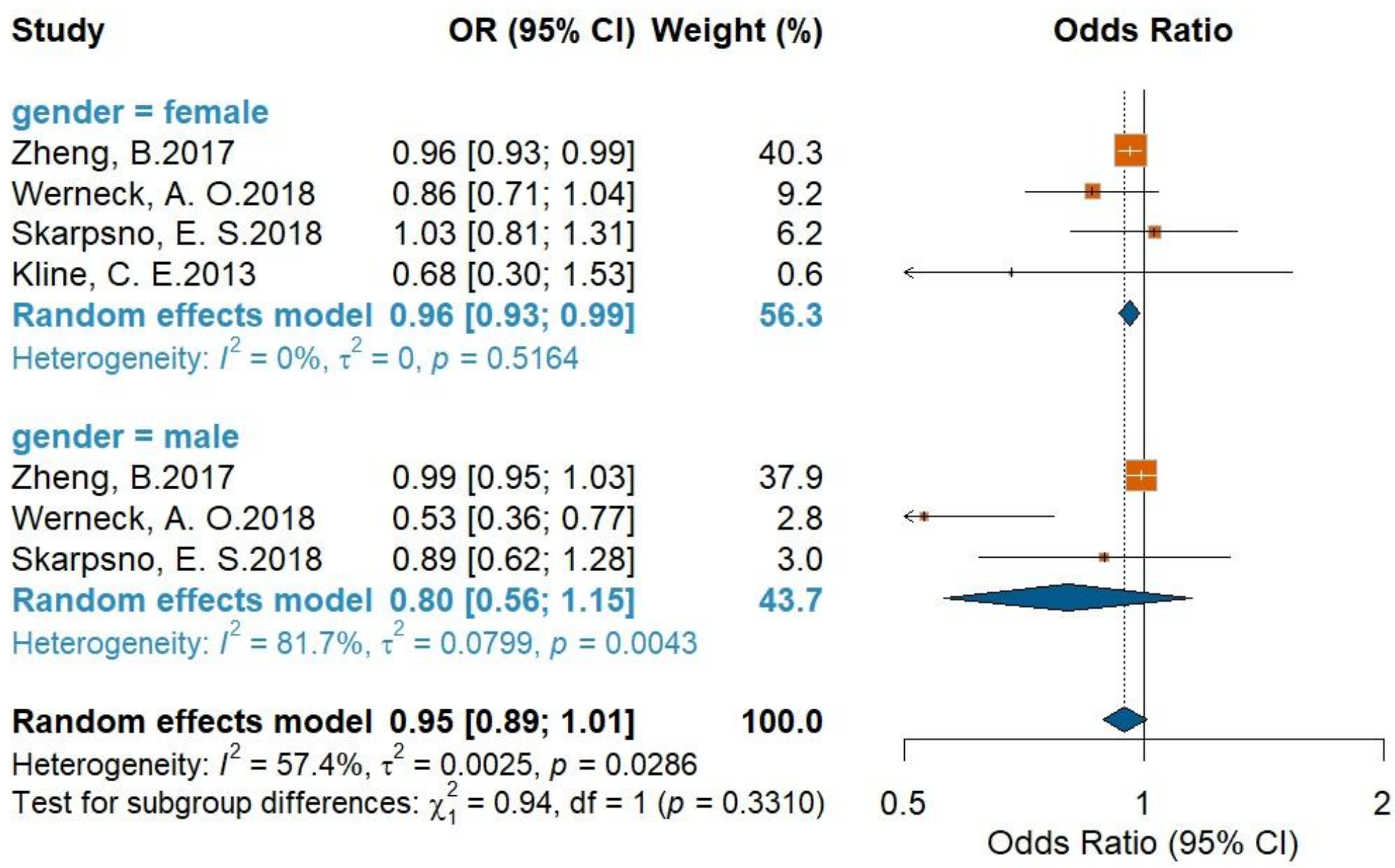

Abbreviations: OR, odds ratio; CI, confidence interval.

Figure S8. Forest plot of a random effects meta-analysis including 27 risk estimates of insomnia for a high versus low level of PA, grouped by PA domain

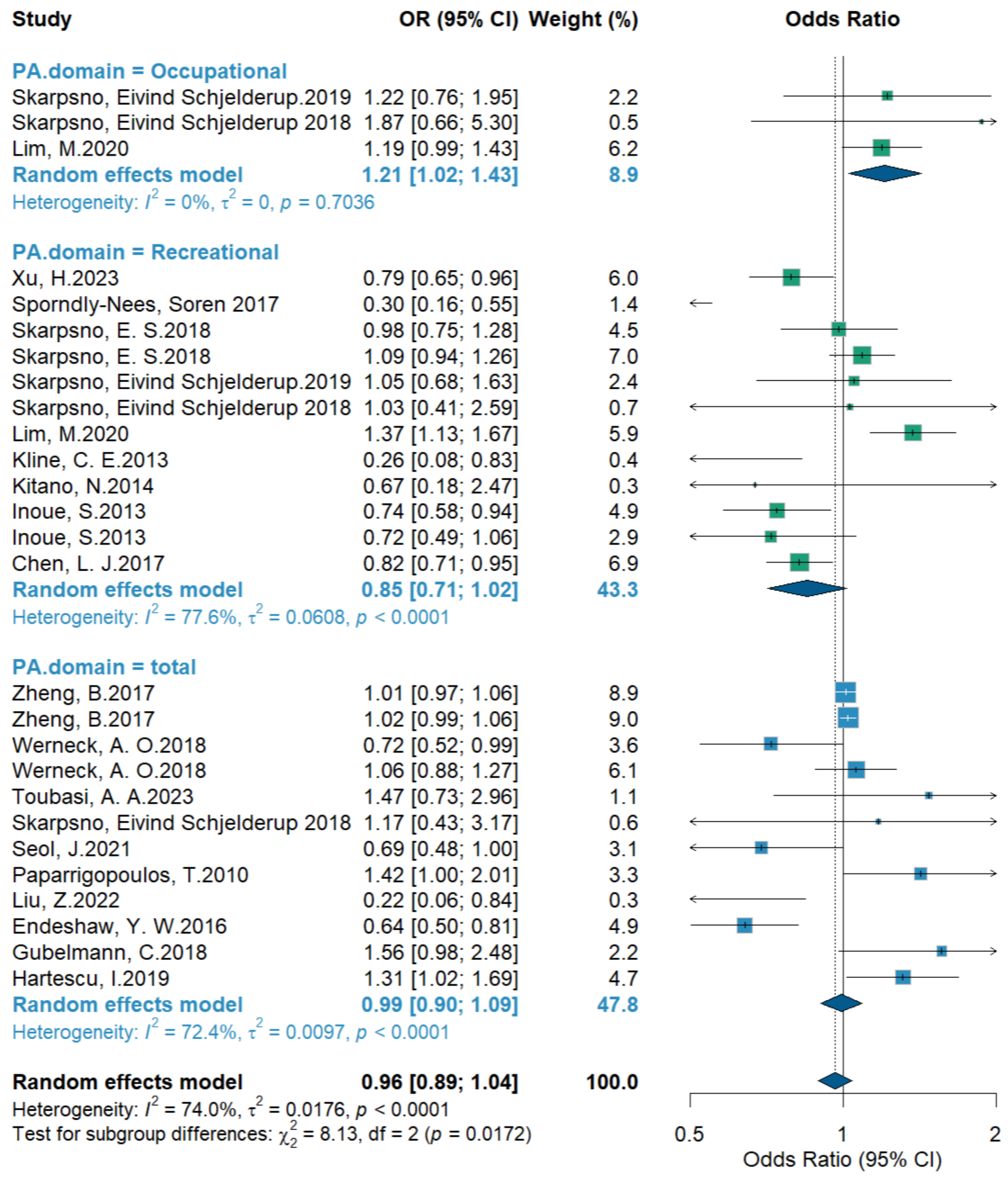

Abbreviations: OR, odds ratio; CI, confidence interval; PA, physical activity.

Figure S9. Forest plot of a random effects meta-analysis including 17 risk estimates of insomnia for a high vs moderate level of PA, grouped by PA domain

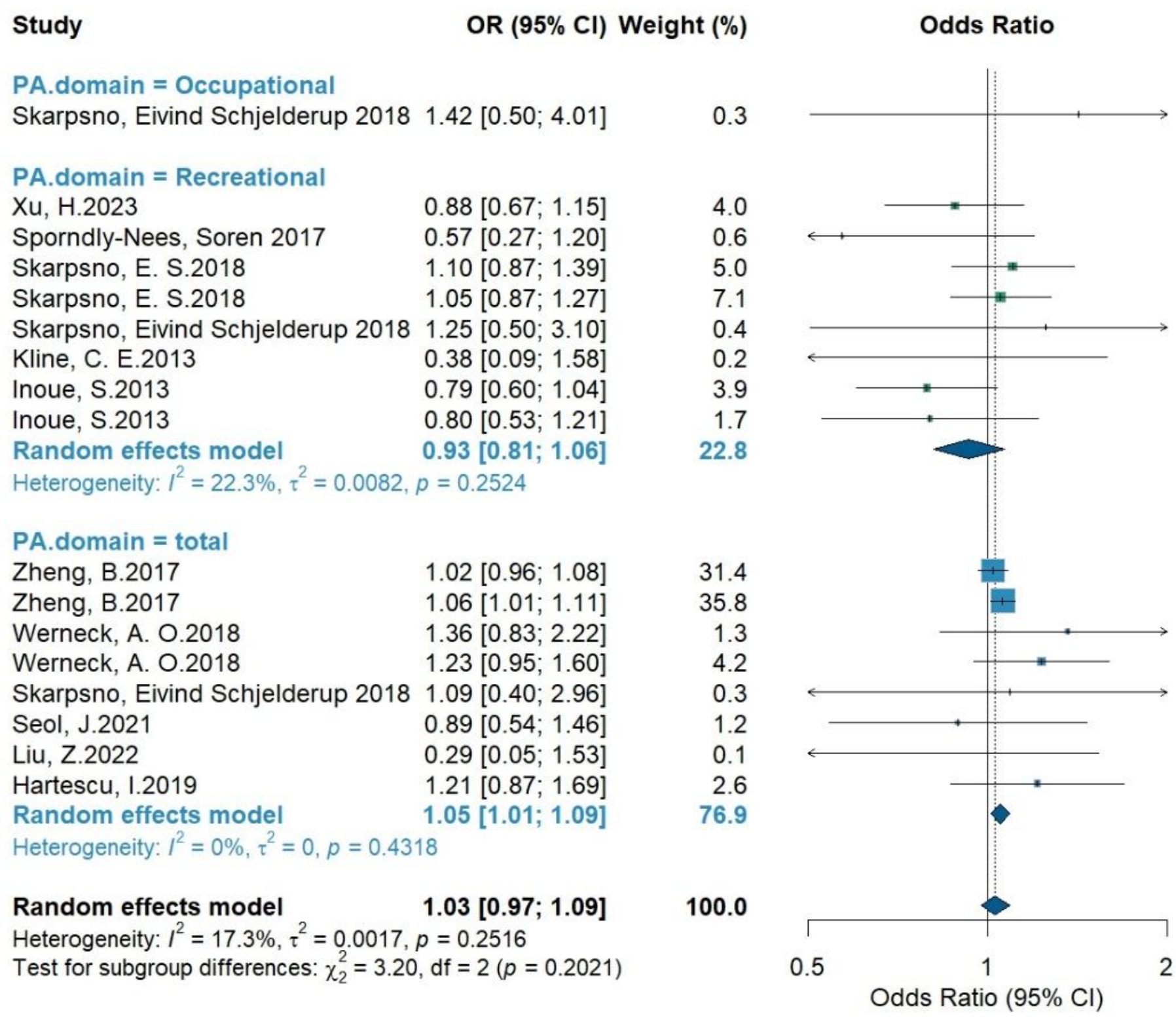

Abbreviations: OR, odds ratio; CI, confidence interval; PA, physical activity.

Figure S10. Forest plot of a random effects meta-analysis including 17 risk estimates of insomnia for a moderate vs low level of PA, grouped by PA domain

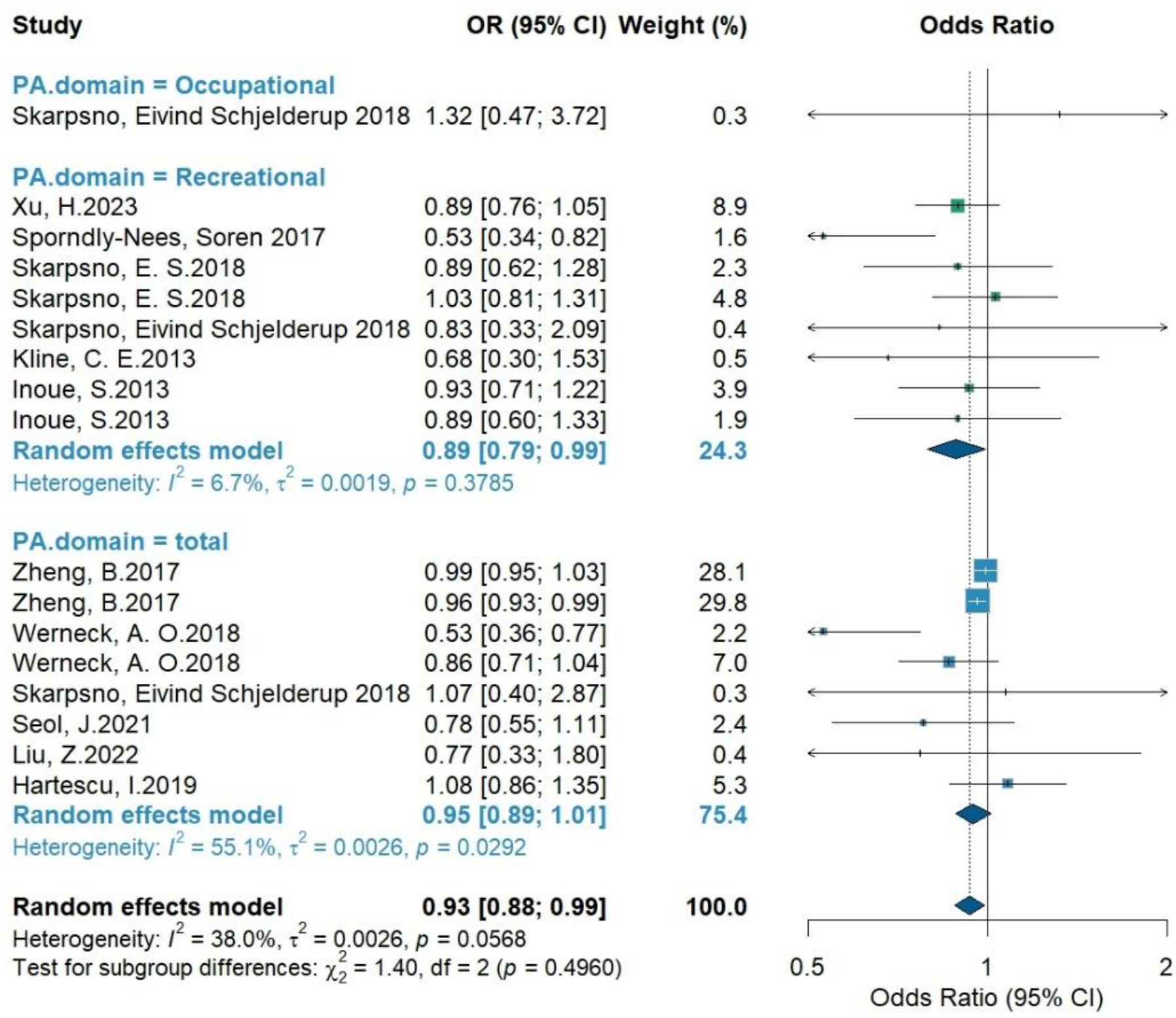

Abbreviations: OR, odds ratio; CI, confidence interval; PA, physical activity.

Figure S11. Leave-One-Out Sensitivity Analysis

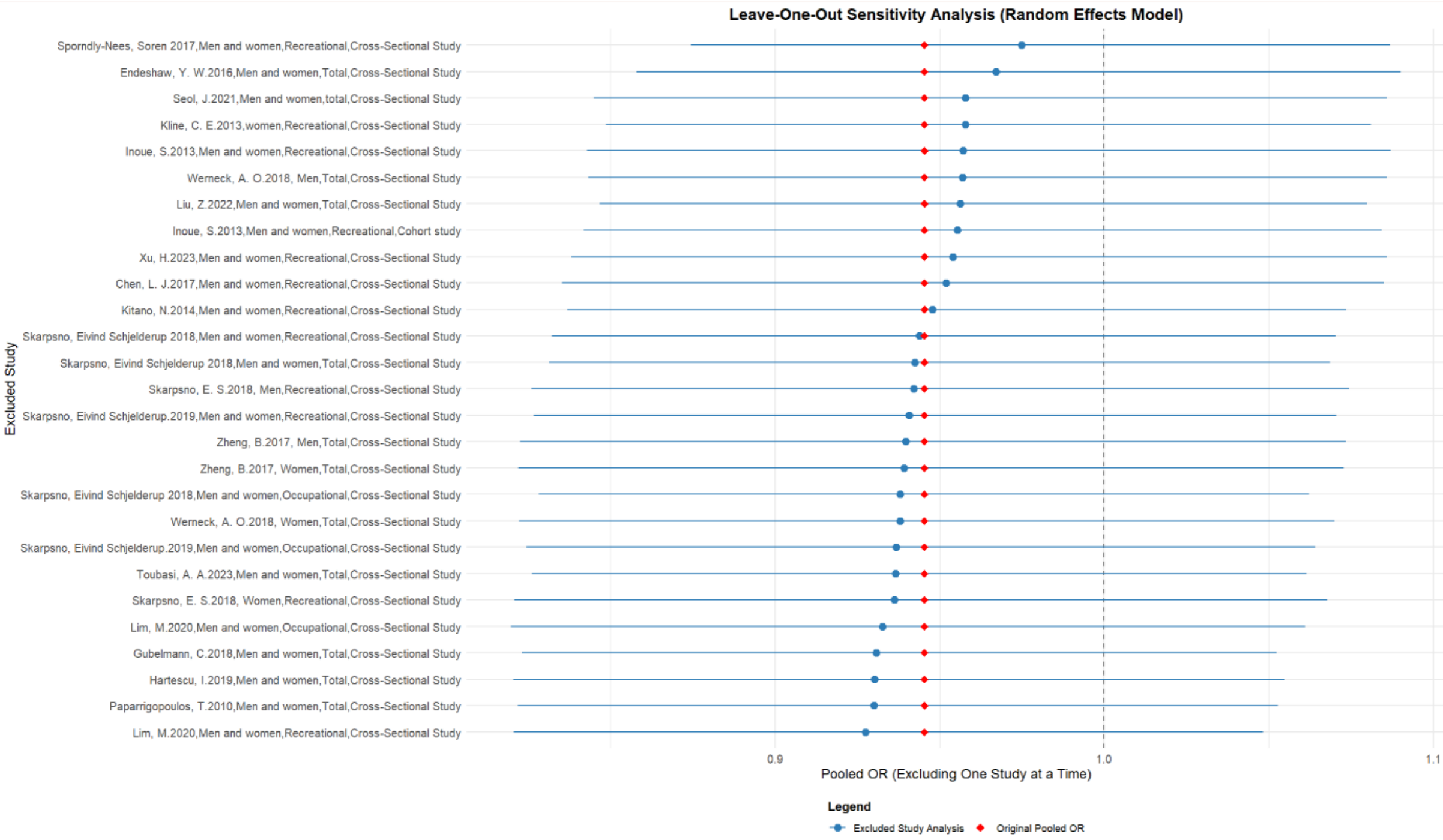

Figure S12. Standardized Funnel plot corresponding to the main random-effects meta-analysis

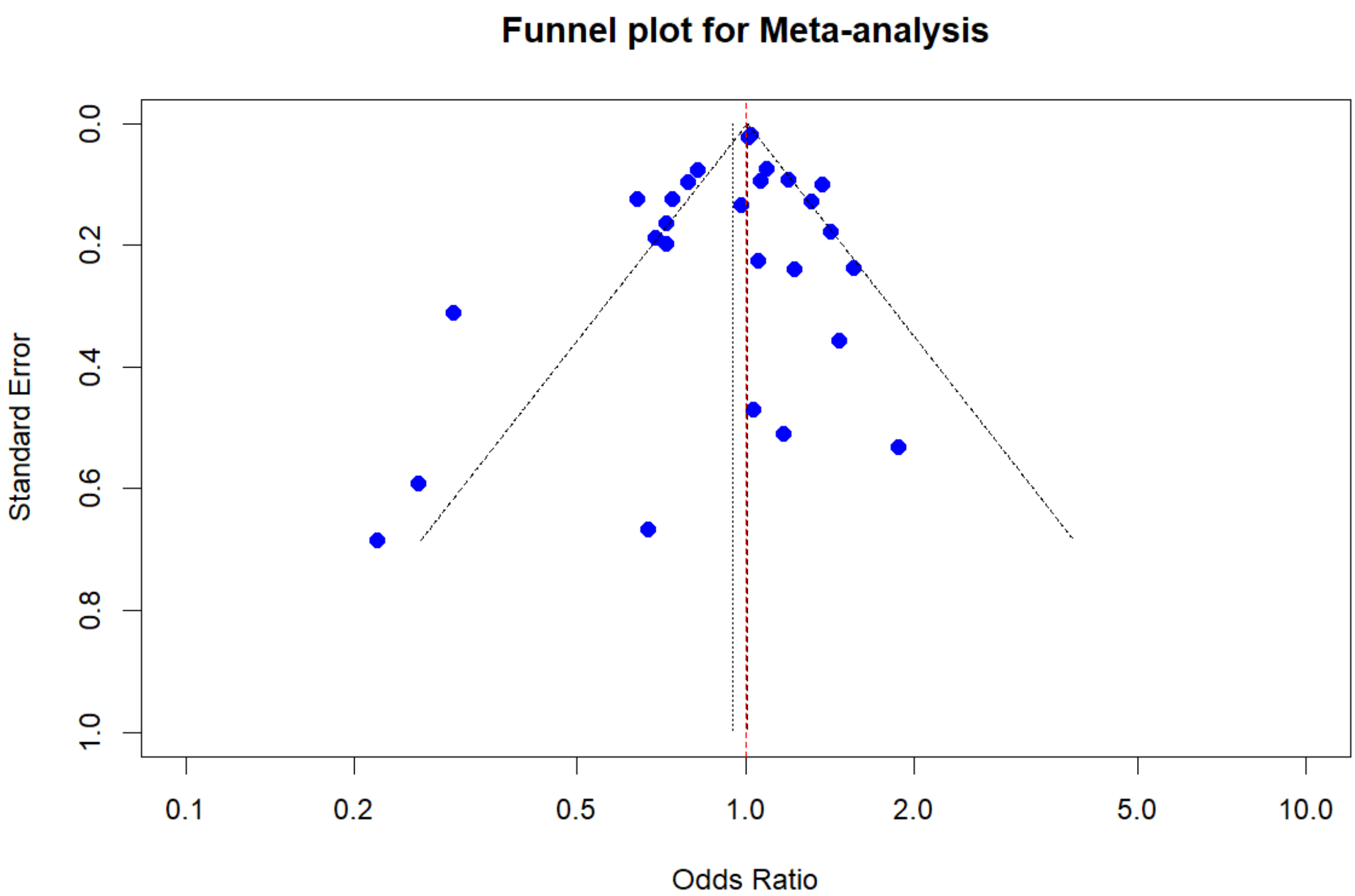

Figure S13. Standardised Begg's regression test corresponding to the main random-effects meta-analysis

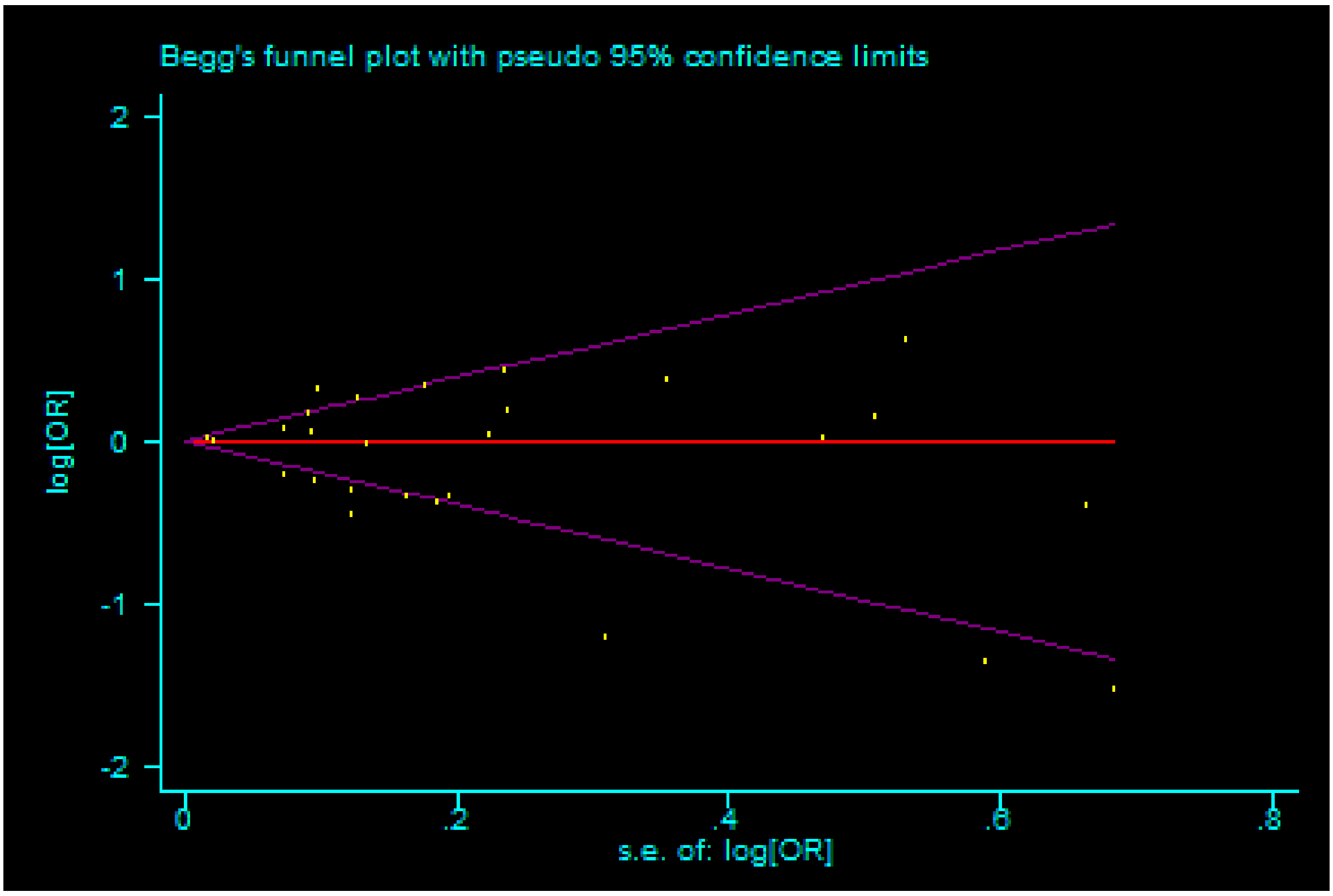

Abbreviations: OR, odds ratio; SE, standard error.

Figure S14. Standardised Egger's regression test corresponding to the main random-effects meta-analysis

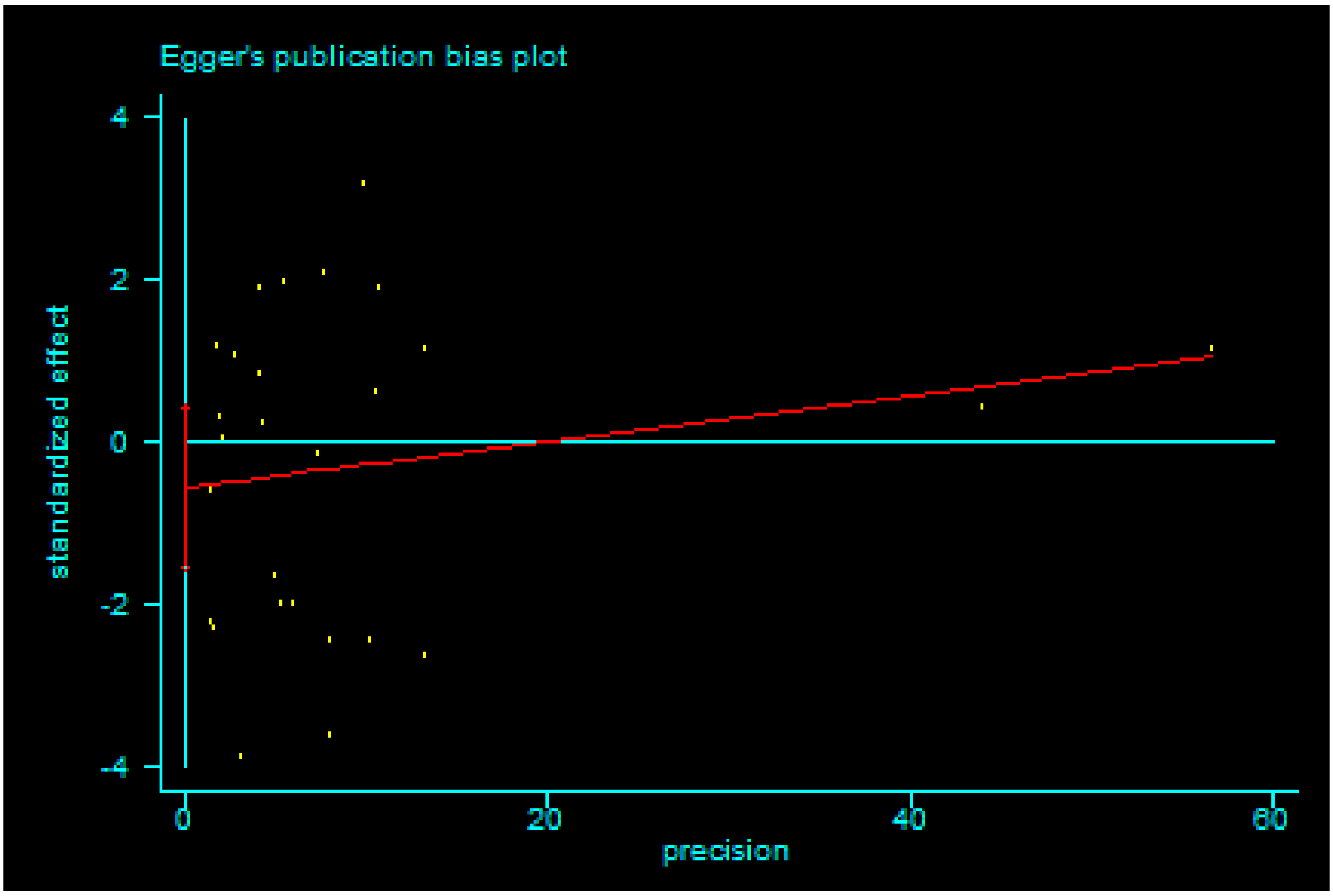

Supplement: Supplementary file 1 [file jogh-16-04196-s001.pdf]
